# Melatonin Improves Salt Tolerance in Tomato Seedlings by Enhancing Photosystem II Functionality and Calvin Cycle Activity

**DOI:** 10.3390/plants14121785

**Published:** 2025-06-11

**Authors:** Xianjun Chen, Bi Chen, Yao Jiang, Jianwei Zhang, Mingjie Liu, Qin Yang, Huiying Liu

**Affiliations:** 1 Provincial Famous Teacher Yang Qin Studio, Key Laboratory of Molecular Breeding and Variety Creation of Horticultural Plants for Mountain Features in Guizhou Province, School of Life and Health Science, Kaili University, Kaili 556011, China; chenxianjun_0805@163.com (X.C.); 18487194930@163.com (B.C.); jiangyao0221@163.com (Y.J.); zhangjw1831@163.com (J.Z.); liumingjiee@163.com (M.L.); 2 Key Laboratory of Special Fruits and Vegetables Cultivation Physiology and Germplasm Resources Utilization of Xinjiang Production and Contruction Crops, Department of Horticulture, Agricultural College, Shihezi University, Shihezi 832003, China

**Keywords:** melatonin, salt stress, tomato seedlings, chlorophyll a fluorescence, Calvin cycle

## Abstract

Salt stress severely impairs photosynthesis and development in tomato seedlings. This study investigated the regulatory role of exogenous melatonin (MT) on photosynthetic performance under salt stress by determining chlorophyll content, chlorophyll a fluorescence parameters, Calvin cycle enzyme activities, and related gene expression. Results showed that salt stress significantly reduced chlorophyll content and impaired photosystem II (PSII) functionality, as evidenced by the increased minimum fluorescence (*F*_o_) and decreased maximum quantum efficiency of PSII (*F*_v_/*F*_m_) and effective PSII quantum yield (*Φ*_PSII_). MT application mitigated these negative effects, as reflected by higher *F*_v_/*F*_m_, increased chlorophyll content, and lower non-photochemical quenching (*NPQ*). In addition, MT-treated plants exhibited improved PSII electron transport and more efficient use of absorbed light energy, as shown by elevated *Φ*_PSII_ and *qP* values. These changes suggest improved PSII functional stability and reduced excess thermal energy dissipation. Furthermore, MT significantly enhanced both the activity and expression of key enzymes involved in the Calvin cycle, including ribulose-1,5-bisphosphate carboxylase/oxygenase (Rubisco), Rubisco activase (RCA), phosphoglycerate kinase (PGK), glyceraldehyde-3-phosphate dehydrogenase (GAPDH), fructose-1,6-bisphosphatase (FBPase), fructose-bisphosphate aldolase (FBA), transketolase (TK), and sedoheptulose-1,7-bisphosphatase (SBPase), thereby promoting carbon fixation and ribulose-1,5-bisphosphate (RuBP) regeneration under salt stress. Conversely, inhibition of endogenous MT synthesis by p-CPA exacerbated salt stress damage, further confirming MT’s crucial role in salt tolerance. These findings demonstrate that exogenous MT enhances salt tolerance in tomato seedlings by simultaneously improving photosynthetic electron transport efficiency and upregulating the activity and gene expression of key Calvin cycle enzymes, thereby promoting the coordination between light reactions and carbon fixation processes. This study provides valuable insights into the comprehensive regulatory role of MT in maintaining photosynthetic performance under saline conditions.

## 1. Introduction

Soil salinization is a major global issue that severely limits vegetable growth and productivity, particularly in facility agriculture [1]. In recent years, the problem of soil salinization in protected cultivation systems has worsened due to factors such as high cropping frequency, excessive fertilization, and insufficient rainfall leaching [2]. This accumulation of salts hinders plant growth and development, leading to reduced crop yield and quality. Consequently, soil salinization poses a critical challenge to the sustainable development of agriculture in China and worldwide. Photosynthesis, a critical physiological process for plant growth, is significantly influenced by environmental stress factors. Elevated salt concentrations in the rhizosphere can exacerbate photoinhibition and photodamage in the photosynthetic apparatus of plants, thereby reducing photosynthetic efficiency [3,4]. In tomato (*Solanum lycopersicum*), a widely cultivated and salt-sensitive vegetable crop, salt stress significantly compromises photosynthetic performance and biomass accumulation, which directly impacts its agricultural productivity [5,6,7]. Therefore, understanding how to mitigate the adverse effects of salt stress on photosynthesis is of great importance for improving tomato salt tolerance and ensuring stable production under saline conditions.

Melatonin (MT), a novel phytohormone or plant growth regulator, significantly enhances plant tolerance to various abiotic stresses [8,9,10]. An expanding corpus of research indicates that MT regulates several physiological and biochemical processes, hence significantly contributing to stress tolerance [11,12,13]. MT treatment has demonstrated the capacity to enhance the tolerance of rice (*Oryza sativa* L.) to salinity and drought by upregulating genes associated with stress responses, reducing the accumulation of reactive oxygen species (ROS), and strengthening the antioxidant defense mechanism [14]. Besides directly scavenging reactive oxygen species (ROS), melatonin (MT), a potent antioxidant, also engages with ROS signaling pathways to jointly control stress responses [15]. An et al. [16] demonstrated that MT improves thermotolerance in tomatoes by promoting the biosynthesis of 5-aminolevulinic acid (ALA), Mg-protoporphyrin (Mg Proto), and protochlorophyllide (Pchlide), while concurrently upregulating genes linked to chlorophyll synthesis (*SlHEMA1*, *SlPORB*, *SlPORC*, and *SlCHLI*) and downregulating genes associated with chlorophyll degradation (*SlCLH1*, *SlCLH2*, *SlPAO*, *SlPPH*, and *SlRCCR*). Transcriptomic studies suggest that exogenous MT may protect rapeseed (*Brassica napus* L.) against salt stress by regulating genes associated with photosynthesis and the ascorbate pathway [17]. Similarly, under cadmium stress, Kumar et al. [18] discovered that MT aids in maintaining the integrity and structure of chloroplasts in the mesophyll cells of sweet potatoes (*Ipomoea batatas* L.). It enhances stomatal density and fortifies the stability of photosynthetic organs and the antioxidant system, thus alleviating the adverse effects of chromium.

Our earlier study showed that exogenous MT administration increases endogenous MT levels by upregulating genes encoding important enzymes in the MT biosynthesis pathway, which in turn improves salt tolerance in tomato seedlings. This increase in MT enhances the safeguarding of the PSII reaction center, optimizes electron transport, and reduces oxidative damage to PSI [19]. These results mostly indicate the photochemical responses of PSII before carbon fixation begins (i.e., prior to the dark reactions), while the application of light energy by the photosynthetic system after the commencement of carbon absorption is inadequately comprehended. The Calvin cycle, as the primary carbon fixation mechanism in photoautotrophs, is essential for transforming inorganic carbon into organic molecules. Improving the carbon fixation efficiency of the Calvin cycle is considered an efficient strategy for augmenting agricultural output [20,21]. Recent studies indicate that MT mitigates photoinhibition in cucumber (*Cucumis sativus* L.) seedlings subjected to low-temperature stress by regulating the Calvin cycle [22], and enhances photosynthetic efficiency in tomato seedlings facing high-temperature stress by elevating the activity of ribulose-1,5-bisphosphate carboxylase/oxygenase (Rubisco) and fructose-1,6-bisphosphatase (FBPase) [23]. Moreover, MT treatment has shown the capacity to enhance the activities of Calvin cycle enzymes and stimulate photosynthesis and yield in wheat (*Triticum aestivum* L.) under salinity stress [24]. Nevertheless, the effect of salt stress on crucial Calvin cycle enzymes in tomato seedlings, together with the role of MT in this context, remains unclear. This study investigated the effects of exogenous MT and its synthesis inhibitor p-chlorophenylalanine (p-CPA) on tomato seedlings during NaCl stress.

## 2. Materials and Methods

### 2.1. Plant Materials and Experimental Treatments

Tomato seedlings (*Solanum lycopersicum* L. cv. ‘Zhongshu No. 4’) were cultivated hydroponically in a temperature- and humidity-controlled greenhouse at Kaili University, Guizhou, China. To promote seedling growth and cotyledon expansion, radicle-emergent seeds were sown in seedling trays filled with a peat-to-vermiculite substrate (2:1, *v*/*v*). Uniform seedlings at the two-leaf, one-heart stage were moved to 12 L black plastic containers, each containing 10 L of Hoagland nutritional solution (pH 6.2) made with deionized water. Treatments were started after a seven-day period of acclimatization in the nutritional solution. Sodium chloride (NaCl) was added to induce salt stress, followed by foliar application of melatonin (MT) and/or p-chlorophenylalanine (p-CPA), an MT biosynthesis inhibitor. The following five treatments were included in the experimental design: (1) Control (no NaCl, MT, or p-CPA), (2) 100 mmol·L^−1^ NaCl (NaCl), (3) 100 mmol·L^−1^ NaCl + 100 μmol·L^−1^ MT (NM), (4) 100 mmol·L^−1^ NaCl + 100 μmol·L^−1^ p-CPA (NP), and (5) 100 mmol·L^−1^ NaCl + 100 μmol·L^−1^ MT + 100 μmol·L^−1^ p-CPA (NMP). MT was procured from Sigma-Aldrich (St. Louis, MO, USA), whereas p-CPA was sourced from Macklin Biochemical Co., Ltd. (Shanghai, China). Four biological replicates were used for each treatment, and five plants were grown in each duplicate. The experiment was set up in a randomized complete block design. Greenhouse conditions were sustained at 24–28 °C during the day and 17–20 °C at night, with a 13 h photoperiod. To guarantee steady nutritional availability, the nutrient solution was changed every two days. At the end of the ninth day of treatment, we took the second completely developed leaf from the apex to analyze later.

### 2.2. Relative Growth Rate

The relative growth rates (RGRs) of plant height, stem diameter, shoot dry weight, and root dry weight were assessed on the ninth day of treatment using the methodology outlined by Tan et al. [25]. The RGR values were computed using the following equations:

$${RGR}_{H}\left(cm\cdot {cm}^{-1}\cdot d^{-1}\right)=\frac{1}{H}\times \frac{dH}{dt}=\frac{lnH_{2}-lnH_{1}}{t_{2}-t_{1}}$$

$${RGR}_{C}\left(mm\cdot {mm}^{-1}\cdot d^{-1}\right)=\frac{1}{C}\times \frac{dC}{dt}=\frac{lnC_{2}-lnC_{1}}{t_{2}-t_{1}}$$

$${RGR}_{R}\left(g\cdot g^{-1}\cdot d^{-1}\right)=\frac{1}{R}\times \frac{dR}{dt}=\frac{lnR_{2}-lnR_{1}}{t_{2}-t_{1}}$$

$${RGR}_{L}\left(g\cdot g^{-1}\cdot d^{-1}\right)=\frac{1}{L}\times \frac{dL}{dt}=\frac{lnL_{2}-lnL_{1}}{t_{2}-t_{1}}$$

In this case, H, C, R, and L stand for plant height (cm), shoot dry weight (g), root dry weight (g), and stem diameter (mm), respectively. Measurements made at the first time point (t_1_, sixth day of treatment) and the last time point (t_2_, ninth day of treatment) of the treatment period are denoted by subscripts 1 and 2.

### 2.3. Chlorophyll Content

Chlorophyll content was assessed from the second completely developed leaf from the apex. Fresh leaf samples (0.5 g) were carefully weighed after thorough washing and blotting, then homogenized in 80% (*v*/*v*) acetone with a tiny quantity of quartz sand to extract photosynthetic pigments. Absorbance values at 663 nm, 645 nm, and 470 nm were recorded at 25 °C using a UV–visible spectrophotometer (UV-1800, Shimadzu, Kyoto, Japan). The contents of chlorophyll a (Chl*a*), chlorophyll b (Chl*b*), and carotenoid (Car) were determined using the methodology outlined by Chen et al. [26]. The total chlorophyll content was represented as Chl*a* + Chl*b*. The chlorophyll *a*/*b* ratio (Chl*a*/Chl*b*) and the carotenoid to total chlorophyll ratio (Car/(Chl*a* + Chl*b*)) were also computed.

### 2.4. Measurement of Chlorophyll a Fluorescence Parameters

Measurements of chlorophyll a fluorescence parameters were performed between 8:00 and 11:00 AM on the ninth day of treatment. The second completely expanded leaf was subjected to a 30 min dark adaptation period before fluorescence parameters were acquired using the Imaging Win application and Maxi Imaging-PAM modulated fluorescence imaging equipment (Imaging-PAM, WALZ, Germany). A modest measuring light (<0.1 μmol·m^−2^·s^−1^) was used to record the lowest fluorescence (*F*_o_), and a strong saturating pulse (8000 μmol·m^−2^·s^−1^) was used to acquire the maximum fluorescence (*F*_m_). All of the fluorescence characteristics were collected after the actinic light (800 μmol·m^−2^·s^−9^) was turned on and the fluorescence signal had stabilized (around three minutes). The instrument automatically provided the following parameters for chlorophyll a fluorescence: PSII electron transport activity (*F*_m_/*F*_o_), photochemical quenching coefficient (*qP*) = (*F*_m_′ − *F*_s_)/(*F*_m_′ − *F*_o_′), actual PSII photochemical efficiency (*Φ*_PSII_) = (*F*_m_′ − *F*_s_)/ *F*_m_′, non-photochemical quenching coefficient (*NPQ*) = (*F*_m_ − *F*_m_′)/*F*_m_′, and maximum photochemical efficiency of PSII (*F*_v_/*F*_m_). The excitation energy distribution coefficients of PSI (*α*) = *f*/(1 + *f*) and PSII (*β*) = 1/(1 + *f*), *f* = (*F*_m_′ − *F*_s_)/(*F*_m_′ − *F*_o_′), the imbalance in excitation energy distribution between PSI and PSII (*β*/*α* − 1) = = (1 − *f*)/*f*, the proportion of absorbed light energy used for photochemical reactions (*P*) = *F*_v_′/*F*_m_′ × *qP* × 100 %, the proportion dissipated as antenna heat (*D*) = (1 − *F*_v_′/*F*_m_′) × 100%, and non-optical dissipation (*Ex*) = *F*_v_′/*F*_m_′ × (1 − *qP*) were calculated using the method of Chen et al. [26,27]. Other calculations included antenna conversion efficiency (*F*_v_′/*F*_m_′), PSII excitation energy pressure (1 − *qP*) = (*F-F*_o_′)/(*F*_m_′-*F*_o_′), photochemical reaction rate (*P*_rate_) = [(*F*_m_′ − *F*_s_)/*F*ₘ′] × PFD, and antenna thermal dissipation rate (*D*_rate_) = (1 − *F*_v_′/*F*_m_′) × PFD. The method suggested by Krall and Edwards [28] was used to calculate the total non-cyclic electron transport rate of PSII (*J*_F_) = *Φ*_PSII_ × PFD × α × *f*, whereas Chen et al. [27] evaluated the relative limitation of photosynthetic function under light circumstances (*L*_PFD_) = 1 − [*qP* × (*F*_m_′ − *F*_s_)/*F*_m_′]/0.83.

### 2.5. Measurement of Key Enzyme Activities in the Calvin Cycle

Using the methodology outlined by Cong et al. [29], the activity of important enzymes involved in the Calvin cycle were measured. The enzymes measured comprised ribulose-1,5-bisphosphate carboxylase/oxygenase (Rubisco, both initial and total activities), phosphoglycerate kinase (PGK), glyceraldehyde-3-phosphate dehydrogenase (GAPDH), fructose-1,6-bisphosphatase (FBPase), fructose-1,6-bisphosphate aldolase (FBA), transketolase (TK), sedoheptulose-1,7-bisphosphatase (SBPase), and Rubisco activase (RCA).

### 2.6. Quantitative Real-Time PCR (qRT-PCR) Analysis

In accordance with the manufacturer’s recommendations, total RNA was extracted from the second completely grown leaf of tomato seedlings using the RNAprep Pure Plant Kit (Tiangen, Beijing, China). A spectrophotometer called the NanoDrop 2000 (Thermo Fisher Scientific, Waltham, MA, USA) was used to measure the extracted RNA’s concentration and purity. Using agarose gel electrophoresis, the integrity of the RNA was evaluated. RNase-free DNase I was used to treat the RNA samples in order to eliminate genomic DNA contamination (Takara, Dalian, China). With the PrimeScript™ RT reagent Kit with gDNA Eraser (Takara, Dalian, China), first-strand cDNA was synthesized in accordance with the manufacturer’s instructions. The TB Green® Premix Ex TaqTM (Takara, Dalian, China) was used in a LightCycler® 96 Real-Time PCR System (Roche, Basel, Switzerland) to conduct quantitative real-time PCR (qRT-PCR). The required primers are shown in Table S1. Relative gene expression levels were determined via the 2^−ΔΔCt^ technique, employing Actin as the internal reference gene. Each sample underwent three biological replicates and three technical repetitions.

### 2.7. Data Analysis

Software called IBM SPSS Statistics 21.0 (IBM Corp., Armonk, NY, USA) was used to conduct statistical analyses. Differences between treatments were evaluated using one-way analysis of variance (ANOVA), and post hoc comparisons were performed using the least significant difference (LSD) test at a significance level of *p* < 0.05. The mean ± standard deviation (Mean ± SD) is used to display all data. Utilizing ORIGIN PRO 9.0 (OriginLab Corp., Northampton, MA, USA), graphs were produced.

## 3. Results

### 3.1. Effects of Melatonin on the Growth of Tomato Seedlings Under Salt Stress

**Figure 1:**
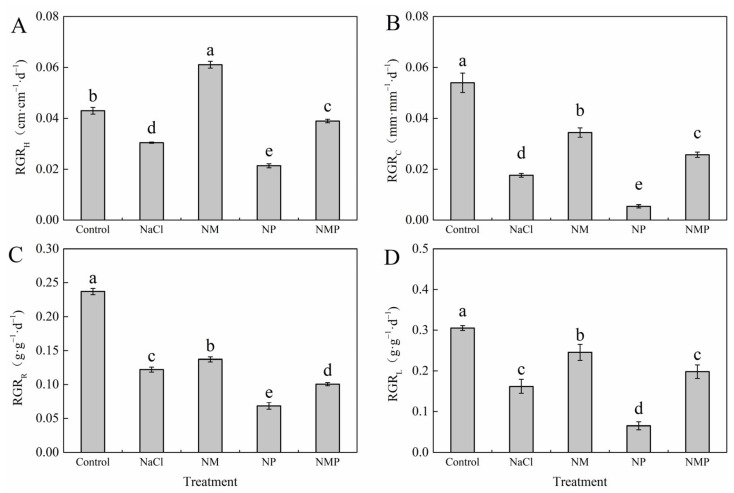
Effects of exogenous melatonin on the relative growth rate of tomato seedlings under salt stress. The relative growth rates of plant height (RGR_H_) (**A**), stem diameter (RGR_C_) (**B**), root dry weight (RGR_R_) (**C**), and shoot dry weight (RGR_L_) (**D**) were measured under different treatments: Control (normal conditions without salt stress), NaCl (100 mM NaCl treatment), NM (100 mM NaCl + 100 μM melatonin), NP (100 mM NaCl + 100 μM p-CPA, a melatonin synthesis inhibitor), and NMP (100 mM NaCl + 100 μM melatonin + 100 μM p-CPA). Different letters above the bars indicate significant differences between treatments (*p* < 0.05) based on one-way ANOVA with LSD multiple comparisons. Values represent means ± standard errors (SE) (*n* = 4).

In tomato seedlings treated with 100 mM NaCl (NaCl), the relative growth rates of plant height (RGR_H_), stem diameter (RGR_C_), shoot dry weight (RGR_L_), and root dry weight (RGR_R_) decreased by 29.1%, 67.4%, 48.6%, and 46.9%, respectively, compared to the Control group (Control, without NaCl stress) (Figure 1). This suggests that tomato seedling development is adversely impacted by NaCl stress. In contrast, these growth indices increased by 100.6%, 95.6%, 12.4%, and 51.6%, respectively, when exogenous 100 μM MT was delivered under NaCl stress (NM treatment). However, the growth-inhibiting effects of salt stress on tomato seedlings were intensified when 100 μM p-chlorophenylalanine (p-CPA, an MT synthesis inhibitor) was applied under NaCl stress (NP treatment). Furthermore, MT’s ability to alleviate salt stress in tomato seedlings was diminished when p-CPA was added.

### 3.2. Effects of Melatonin on Photosynthetic Pigments in Tomato Seedlings Under Salt Stress

**Figure 2:**
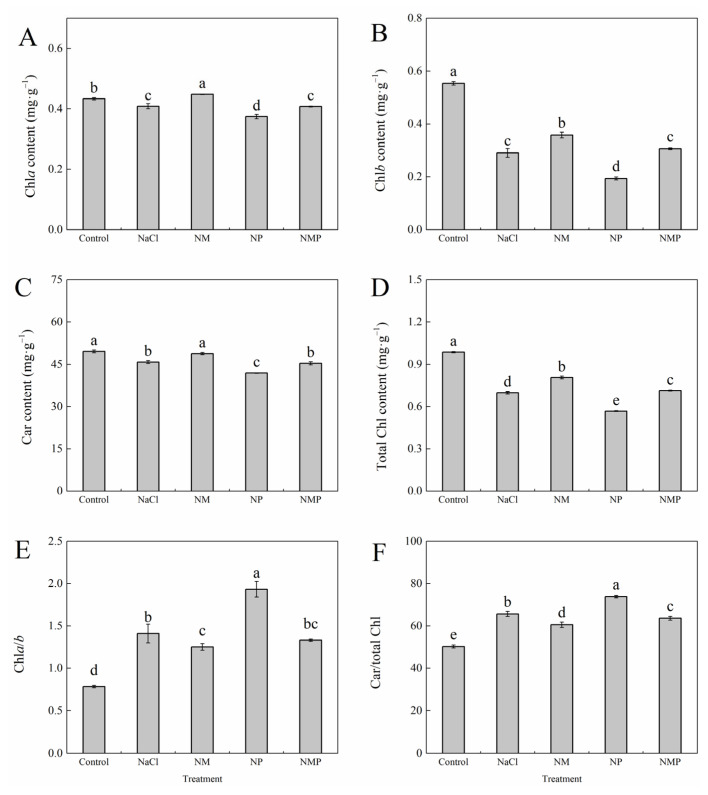
Effects of exogenous melatonin on photosynthetic pigment content in tomato seedlings under salt stress. The contents of chlorophyll a (Chl*a*) (**A**), chlorophyll b (Chl*b*) (**B**), carotenoids (Car) (**C**), and total chlorophyll (Total Chl) (**D**), the Chl*a*/*b* ratio (**E**), and the Car/total Chl ratio (**F**) were measured under different treatments: Control (normal conditions without salt stress), NaCl (100 mM NaCl treatment), NM (100 mM NaCl + 100 μM melatonin), NP (100 mM NaCl + 100 μM p-CPA, a melatonin synthesis inhibitor), and NMP (100 mM NaCl + 100 μM melatonin + 100 μM p-CPA). Different letters above the bars indicate significant differences between treatments (*p* < 0.05) based on one-way ANOVA with LSD multiple comparisons. Values represent means ± standard errors (SE) (*n* = 4).

Figure 2 illustrates that, in comparison to the Control, NaCl treatment markedly decreased the amounts of chlorophyll a (Chl*a*), chlorophyll b (Chl*b*), carotenoid (Car), and total chlorophyll (total Chl), while dramatically elevating the Chl*a*/*b* ratio and the Car/total Chl ratio. This indicates that salt stress impairs chlorophyll production and alters pigment equilibrium in tomato seedlings. The adverse effects of salt stress were mitigated by the NM treatment (NaCl + exogenous MT), which resulted in notable increases of 9.7% in Chl*a*, 23.4% in Chl*b*, 6.5% in Car, and 15.4% in total Chl content. At the same time, the Chl*a*/*b* ratio was significantly reduced by 11.3%, and the Car/total Chl ratio was significantly reduced by 7.8%. Nevertheless, the reduction in photosynthetic pigment content caused by NaCl stress was further exacerbated by the application of p-CPA under salt stress (NP treatment). The NP treatment considerably increased the Chl*a*/*b* ratio by 37.0% and the Car/total Chl ratio by 12.5%, while significantly decreasing the Chl*a*, Chl*b*, Car, and total Chl contents by 8.3%, 33.2%, 8.5%, and 18.7%, respectively, in comparison to the NaCl-only treatment. Furthermore, the effects of exogenous melatonin on these parameters were dramatically negated by the NMP treatment (MT + p-CPA under salt stress) in comparison to the NM treatment.

### 3.3. Effects of Melatonin on Dark-Adapted Chlorophyll a Fluorescence Parameters in Tomato Seedlings Under Salt Stress

**Figure 3:**
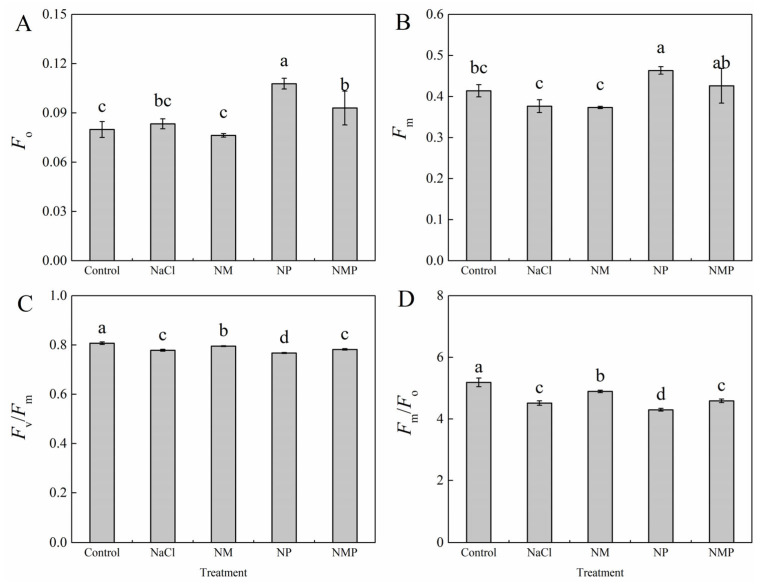
Effects of exogenous melatonin on dark-adapted chlorophyll a fluorescence parameters of tomato seedlings under salt stress. The initial fluorescence (*F*_o_) (**A**), maximum fluorescence (*F*_m_) (**B**), maximum quantum efficiency of photosystem II (*F*_v_/*F*_m_) (**C**), and electron transport activity (*F*_m_/*F*_o_) (**D**) were measured under different treatments: Control (normal conditions without salt stress), NaCl (100 mM NaCl treatment), NM (100 mM NaCl + 100 μM melatonin), NP (100 mM NaCl + 100 μM p-CPA, a melatonin synthesis inhibitor), and NMP (100 mM NaCl + 100 μM melatonin + 100 μM p-CPA). Different letters above the bars indicate significant differences between treatments (*p* < 0.05) based on one-way ANOVA with LSD multiple comparisons. Values represent means ± standard errors (SE) (*n* = 4).

Although it was not statistically significant, the early fluorescence (*F*_o_) in tomato seedling leaves under NaCl stress increased somewhat when compared to the Control group. Maximum fluorescence (*F*_m_) decreased with NaCl exposure by the ninth day of treatment, while the decline was not statistically significant. NaCl stress, however, markedly decreased electron transport activity (*F*_m_/*F*_o_) and photosystem II’s maximal quantum efficiency (*F*_v_/*F*_m_), suggesting compromised PSII function (Figure 3). When compared to the NaCl treatment, the administration of exogenous MT (NM treatment) substantially enhanced *F*_v_/*F*_m_ by 2.2% and *F*_m_/*F*_o_ by 8.4%, but had no discernible impact on *F*_o_ or *F*_m_. This suggests that melatonin may have lessened the negative effects of salt stress on PSII efficiency. On the other hand, the harm brought on by NaCl stress was made worse by the suppression of melatonin production by p-CPA (NP treatment), which resulted in a notable rise of 29.3% in *F*_o_ and 23.1% in *F*_m_, as well as a further decrease in *F*_v_/*F*_m_ by 1.4% and *F*_m_/*F*_o_ by 7.8%. Additionally, the NMP treatment dramatically counteracted the beneficial effects of melatonin on these fluorescence characteristics as compared to the NM treatment.

### 3.4. Effects of Melatonin on Light-Adapted Chlorophyll a Fluorescence Parameters in Tomato Seedlings Under Salt Stress

**Figure 4:**
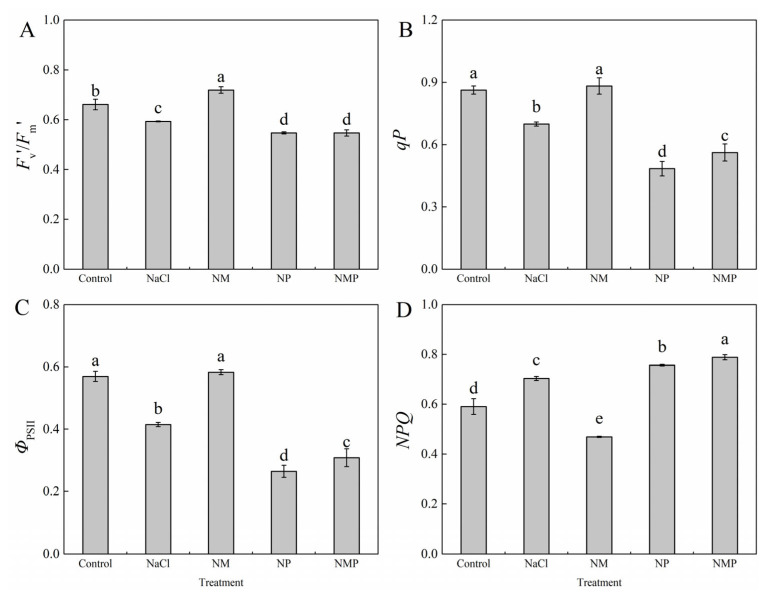
Effects of exogenous melatonin on light-adapted chlorophyll a fluorescence parameters of tomato seedlings under salt stress. The antenna conversion efficiency (*F*_v_′/*F*_m_′) (**A**), the photochemical quenching coefficient (*qP*) (**B**), the actual photochemical efficiency of PSII (*Φ*_PSII_) (**C**), and the non-photochemical quenching coefficient (*NPQ*) (**D**) were measured under different treatments: Control (normal conditions without salt stress), NaCl (100 mM NaCl treatment), NM (100 mM NaCl + 100 μM melatonin), NP (100 mM NaCl + 100 μM p-CPA, a melatonin synthesis inhibitor), and NMP (100 mM NaCl + 100 μM melatonin + 100 μM p-CPA). Different letters above the bars indicate significant differences between treatments (*p* < 0.05) based on one-way ANOVA with LSD multiple comparisons. Values represent means ± standard errors (SE) (*n* = 4).

NaCl stress enhanced the non-photochemical quenching coefficient (*NPQ*) by 19.1% while significantly decreasing the antenna conversion efficiency (*F*_v_′/*F*_m_′), actual photochemical efficiency of PSII (*Φ*_PSII_), and photochemical quenching coefficient (*qP*) by 10.2%, 27.2%, and 18.9%, respectively, in comparison to the Control group (Figure 4). By dramatically increasing *F*_v_′/*F*_m_′, *Φ*_PSII_, and *qP* and significantly decreasing *NPQ* under salt stress conditions, the use of exogenous MT (NM treatment) mitigated these negative effects. In contrast, the detrimental effects of NaCl stress were further intensified by blocking melatonin production with p-CPA (NP treatment), which resulted in a marked rise in *NPQ* and a considerable decrease in *F*_v_′/*F*_m_′, *Φ*_PSII_, and *qP*. Furthermore, the NMP treatment dramatically increased *NPQ* by 68.0% while reducing *F*_v_′/*F*_m_′ by 23.9%, *Φ*_PSII_ by 47.1%, and *qP* by 36.3% in comparison to the NM treatment.

### 3.5. Effects of Melatonin on the Distribution of Excitation Energy Between PSI and PSII in Tomato Seedlings Under Salt Stress

**Figure 5:**
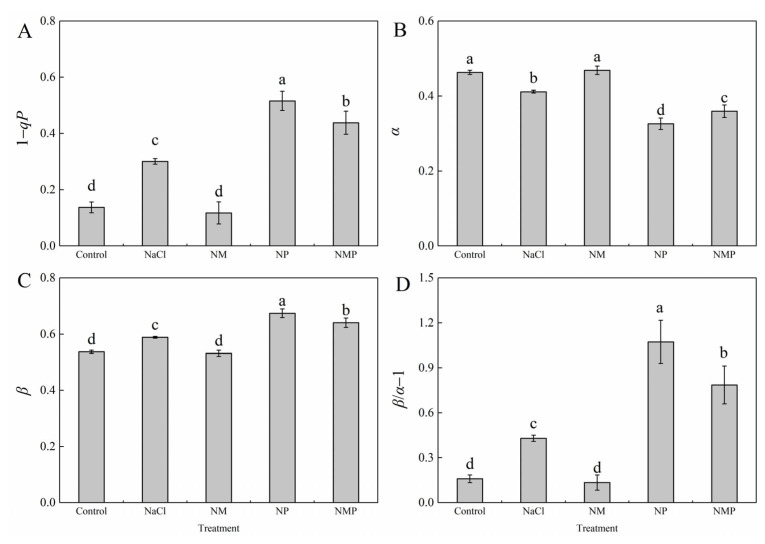
Effects of exogenous melatonin on the distribution of excitation energy between PSI and PSII of tomato seedlings under salt stress. The PSII excitation energy pressure (1 − *qP*) (**A**), PSI excitation energy allocation coefficient (*α*) (**B**), the PSII excitation energy allocation coefficient (*β*) (**C**), and the imbalance deviation coefficient of excitation energy distribution between PSI and PSII (*β*/*α* − 1) (**D**) were measured under different treatments: Control (normal conditions without salt stress), NaCl (100 mM NaCl treatment), NM (100 mM NaCl + 100 μM melatonin), NP (100 mM NaCl + 100 μM p-CPA, a melatonin synthesis inhibitor), and NMP (100 mM NaCl + 100 μM melatonin + 100 μM p-CPA). Different letters above the bars indicate significant differences between treatments (*p* < 0.05) based on one-way ANOVA with LSD multiple comparisons. Values represent means ± standard errors (SE) (*n* = 4).

Figure 5 illustrates that the PSII excitation energy pressure (1 − *qP*), the PSII excitation energy allocation coefficient (*β*), and the imbalance deviation coefficient of excitation energy distribution between PSI and PSII (*β*/*α* − 1) were all significantly increased by salt stress induced by NaCl, whereas the PSI excitation energy allocation coefficient (*α*) was significantly decreased. With the use of exogenous MT (NM) administration, these effects were successfully counteracted, resulting in a noteworthy 13.9% rise in *α*, a 60.9% drop in 1 − *qP*, a 9.7% decrease in *β*, and a 68.6% reduction in *β*/*α* − 1. But the consequences of salt stress were made worse by p-CPA (NP treatment), which inhibited MT production. This led to a notable decrease in *α* and a considerable rise in 1 − *qP*, *β*, and *β*/*α* − 1. These positive effects were undone by the NMP treatment in contrast to the NM treatment.

### 3.6. Effects of Melatonin on the Non-Cyclic Electron Transport Rate, Photochemical Reaction Rate, Thermal Dissipation Rate, and Photosynthetic Function Limitation in Tomato Seedlings Under Salt Stress

**Figure 6:**
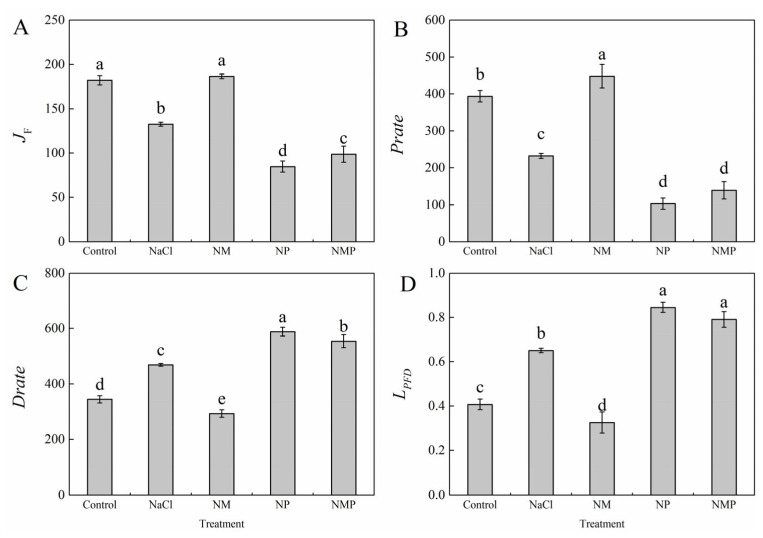
Effects of exogenous melatonin on the non-cyclic electron transport rate (*J*_F_) (**A**), the photochemical reaction rate (*P*_rate_) (**B**), the thermal dissipation rate (*D*_rate_) (**C**), and the relative limitation of photosynthetic function (*L*_PFD_) (**D**) of tomato seedlings under salt stress. Control (normal conditions without salt stress), NaCl (100 mM NaCl treatment), NM (100 mM NaCl + 100 μM melatonin), NP (100 mM NaCl + 100 μM p-CPA, a melatonin synthesis inhibitor), and NMP (100 mM NaCl + 100 μM melatonin + 100 μM p-CPA). Different letters above the bars indicate significant differences between treatments (*p* < 0.05) based on one-way ANOVA with LSD multiple comparisons. Values represent means ± standard errors (SE) (*n* = 4).

NaCl stress raised the thermal dissipation rate (*D*_rate_) and the relative limitation of photosynthetic function (*L*_PFD_) by 36.1% and 59.7%, respectively, on the ninth day of treatment, while significantly decreasing the non-cyclic electron transport rate (*J*_F_) and the photochemical reaction rate (*P*_rate_) by 27.2% and 41.0%, respectively (Figure 6). Application of exogenous MT (NM treatment) reduced *D*_rate_ and *L*_PFD_ while dramatically increasing *J*_F_ and *P*_rate_ as compared to NaCl treatment. In contrast, *J*_F_ and *P*_rate_ were further repressed and the elevations in *D*_rate_ and *L*_PFD_ were made worse by the reduction in melatonin production with p-CPA (NP treatment). NMP treatment resulted in a 47.1% drop in *J*_F_, a 69.0% decrease in *P*_rate_, an 89.2% rise in *D*_rate_, and a 143.0% increase in *L*_PFD_ in comparison to NM treatment.

### 3.7. Effects of Melatonin on the Distribution of Absorbed Light Energy in PSII of Tomato Seedlings Under Salt Stress

**Figure 7:**
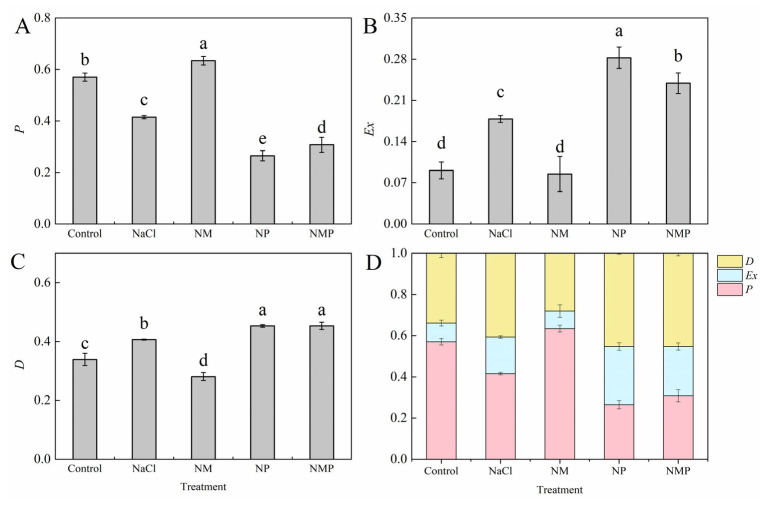
Effects of exogenous melatonin on the distribution of absorbed light energy in PSII of tomato seedlings under salt stress. The proportion of absorbed light energy allocated to photochemical reactions (*P*) (**A**), the energy dissipated through non-photochemical quenching at the P680 reaction center (*Ex*) (**B**), antenna thermal dissipation (*D*) (**C**), and *P* + *Ex* + *D* = 1 (**D**) were measured under different treatments: Control (normal conditions without salt stress), NaCl (100 mM NaCl treatment), NM (100 mM NaCl + 100 μM melatonin), NP (100 mM NaCl + 100 μM p-CPA, a melatonin synthesis inhibitor), and NMP (100 mM NaCl + 100 μM melatonin + 100 μM p-CPA). Different letters above the bars indicate significant differences between treatments (*p* < 0.05) based on one-way ANOVA with LSD multiple comparisons. Values represent means ± standard errors (SE) (*n* = 4).

The percentage of absorbed light energy in PSII that was devoted to photochemical reactions (*P*) was considerably decreased under NaCl stress, whereas the energy dissipated by antenna thermal dissipation (*D*) and non-photochemical quenching at the P680 reaction center (*Ex*) was significantly increased (Figure 7). *P* was considerably raised by 52.8% while *Ex* and *D* were decreased by 52.4% and 30.9%, respectively, by exogenous MT administration (NM treatment). The restriction of melatonin production with p-CPA (NP treatment) intensified the effects of salt stress, resulting in a 36.2% reduction in *P*, a 58.2% rise in *Ex*, and an 11.4% increase in *D*. In comparison to NM treatment, NMP treatment markedly decreased *P* while further enhancing *Ex* and *D*.

### 3.8. Effects of Melatonin on the Activity and Gene Expression of Key Enzymes in the Calvin Cycle of Tomato Seedlings Under Salt Stress

**Figure 8:**
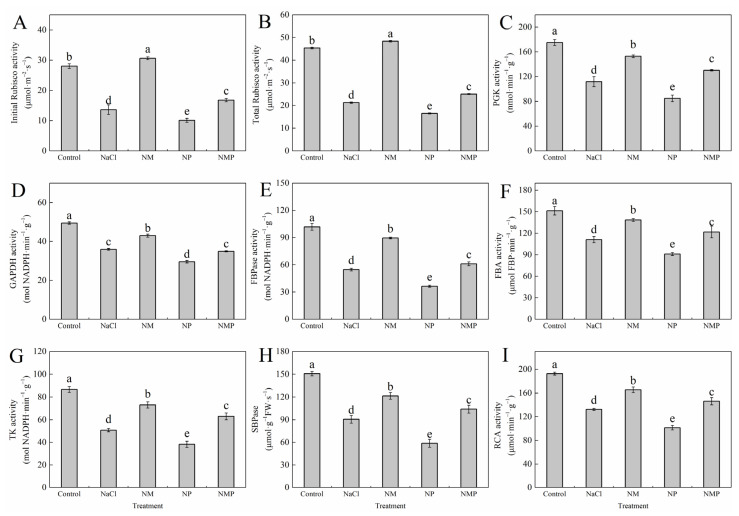
Effects of exogenous melatonin on the activity of key enzymes in the Calvin cycle of tomato seedlings under salt stress. The initial Rubisco activity (**A**), total Rubisco activity (**B**), phosphoglycerate kinase (PGK) (**C**), glyceraldehyde-3-phosphate dehydrogenase (GAPDH) (**D**), fructose-1,6-bisphosphatase (FBPase) (**E**), fructose-1,6-bisphosphate aldolase (FBA) (**F**), transketolase (TK) (**G**), sedoheptulose-1,7-bisphosphatase (SBPase) (**H**), and Rubisco activase (RCA) (**I**) were measured under different treatments: Control (normal conditions without salt stress), NaCl (100 mM NaCl treatment), NM (100 mM NaCl + 100 μM melatonin), NP (100 mM NaCl + 100 μM p-CPA, a melatonin synthesis inhibitor), and NMP (100 mM NaCl + 100 μM melatonin + 100 μM p-CPA). Different letters above the bars indicate significant differences between treatments (*p* < 0.05) based on one-way ANOVA with LSD multiple comparisons. Values represent means ± standard errors (SE) (*n* = 4).

Figure 8 illustrates that NaCl stress markedly diminished the activities of essential Calvin cycle enzymes in the leaves of tomato seedlings, including ribulose-1,5-bisphosphate carboxylase/oxygenase (Rubisco, both initial and total activity), phosphoglycerate kinase (PGK), glyceraldehyde-3-phosphate dehydrogenase (GAPDH), fructose-1,6-bisphosphatase (FBPase), fructose-1,6-bisphosphate aldolase (FBA), transketolase (TK), sedoheptulose-1,7-bisphosphatase (SBPase), and Rubisco activase (RCA). But the activities of these enzymes were greatly enhanced by exogenous MT (NM) administration; the initial Rubisco activity rose by 124.8%, the total Rubisco activity by 127.4%, the PGK by 36.7%, the GAPDH by 19.5%, the FBPase by 63.6%, the FBA by 24.8%, the TK by 43.8%, the SBPase by 34.1%, and the RCA by 25.0%. In contrast, by dramatically reducing the activity of these enzymes, the administration of p-CPA (NP treatment) worsened the inhibitory effects of salt stress. Total Rubisco activity, PGK, GAPDH, FBPase, FBA, TK, SBPase, RCA, and beginning Rubisco activity were significantly decreased with NMP therapy as opposed to NM treatment.

**Figure 9:**
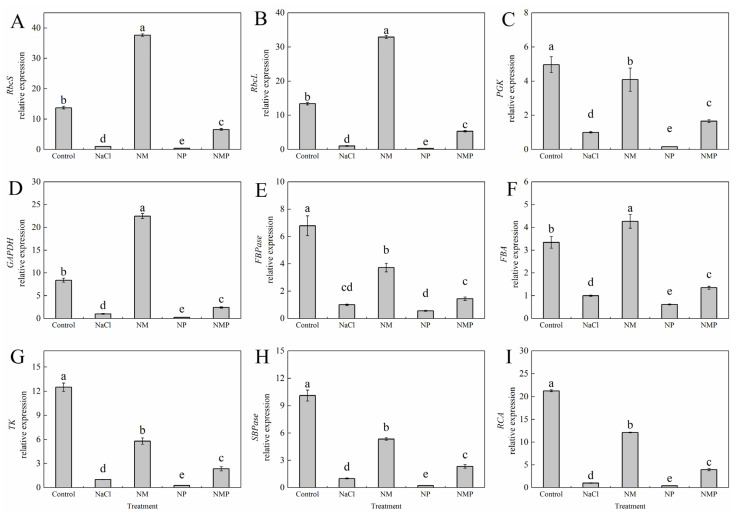
Effects of exogenous melatonin on the gene expression of key enzymes in the Calvin cycle of tomato seedlings under salt stress. The gene expression of *RbcS* (**A**), *RbcL* (**B**), *PGK* (**C**), *GAPDH* (**D**), *FBPase* (**E**), *FBA* (**F**), *TK* (**G**), *SBPase* (**H**), and *RCA* (**I**) were measured under different treatments: Control (normal conditions without salt stress), NaCl (100 mM NaCl treatment), NM (100 mM NaCl + 100 μM melatonin), NP (100 mM NaCl + 100 μM p-CPA, a melatonin synthesis inhibitor), and NMP (100 mM NaCl + 100 μM melatonin + 100 μM p-CPA). Different letters above the bars indicate significant differences between treatments (*p* < 0.05) based on one-way ANOVA with LSD multiple comparisons. Values represent means ± standard errors (SE) (*n* = 4).

NaCl stress significantly diminished enzyme activity and substantially downregulated the expression of essential Calvin cycle genes, with reductions of 92.7-fold in *RbcS*, 92.5-fold in *RbcL*, 79.8-fold in *PGK*, 88.0-fold in *GAPDH*, 85.2-fold in *FBP*, 70.0-fold in *FBA*, 92.0-fold in *TK*, 90.1-fold in *SBP*, and 95.3-fold in *RCA* (Figure 9). However, during salt stress, p-CPA administration inhibited the expression of these genes, whereas exogenous MT markedly increased their expression levels. These tendencies were considerably reversible with NMP treatment as opposed to NM treatment.

## 4. Discussion

The conversion of light energy into chemical energy is accomplished via photosynthesis, one of the most essential and basic biological processes. Photosynthesis is unquestionably a major factor in crop output, but it is very sensitive to changes in the environment [30,31,32]. Numerous studies have shown that salt stress significantly reduces photosynthesis and plant development, which makes it difficult to produce vegetables [33,34,35]. According to our data, salt stress considerably decreased the relative growth rate of tomato seedlings (GRG_H_, GRG_C_, RGR_L_, RGR_R_) (Figure 1), while exogenous melatonin (MT) successfully counteracted these negative effects. This supports earlier research that found MT mitigates the negative effects of salt stress on the buildup of plant biomass [14,36,37]. The chloroplast is very vulnerable to a variety of environmental stressors, which limit the performance of the photosystems and ultimately lower crop production since it is the organelle where photosynthesis occurs. A leaf’s ability to photosynthesize is directly related to the amount of chlorophylls, which are the main pigments that absorb light energy [38]. In this work, tomato seedling leaves under salt stress showed a significant decrease in total chlorophyll content (Figure 2), whereas the Chl *a*/*b* ratio increased. The antenna complexes of photosystem II are the primary location of chlorophyll b (Chl *b*), which is more prone to degradation under stress, according to Shahzadi [39]. The majority of chlorophyll a (Chl *a*) is found in the reaction centers, and it is somewhat more stable. Under salt stress, this differential degradation causes the Chl *a*/*b* ratio to rise. An essential part of the light-harvesting complex, carotenoids (Car) are vital antioxidants. By scavenging ROS produced under stressful situations and squelching surplus energy, they successfully safeguard the photosynthetic machinery [40]. Carotenoids have been shown to decay more slowly than total chlorophyll (Chl *a* + *b*) under environmental stress, leading to an elevated Car/Chl *a* + *b* ratio. This suggests that the plant is responding defensively [41]. It has been shown that MT has substantial protective effects on chlorophyll content [42,43]. According to Figure 2 of our investigation, foliar MT administration reduced the salt-induced chlorophyll degradation, suggesting that MT may encourage chlorophyll production while postponing its breakdown. The p-CPA (NP) treatment group had the lowest Car content. Prior research indicates that p-CPA markedly diminishes endogenous MT levels [19], implying that reduced MT concentrations hinder both light energy conversion efficiency and ROS-scavenging ability. In contrast, exogenous MT successfully maintains the integrity of the photosynthetic machinery under stress conditions [44].

Research has shown that the xanthophyll cycle-mediated non-radiative energy dissipation often results in a drop in minimum fluorescence (*F*_o_) [45,46]. However, photoinhibition may happen when photoprotective systems are not enough to disperse excess light energy. This can lead to damage or the reversible inactivation of PSII reaction centers, which is usually linked to a rise in *F*_o_ [47]. The *F*_m_/*F*_o_ ratio is often used as an indicator of the potential photochemical activity of PSII reaction centers, whereas the *F*_v_/*F*_m_ value represents the maximum quantum efficiency of PSII photochemistry under dark-adapted conditions. One characteristic of photoinhibition is a decrease in *F*_v_/*F*_m_, and damage to PSII response centers is further indicated by a concurrent rise in *F*_o_ [48]. In this investigation, tomato seedlings under salt stress had significant photoinhibition, which was made worse by the suppression of endogenous MT production (Figure 3). Conversely, the exogenous administration of MT significantly mitigated the inhibition and impairment of PSII under saline stress, consequently augmenting both light energy conversion efficiency and electron transport activity via PSII, eventually boosting salt tolerance in tomato plants. In response to changing light conditions, the thylakoid membrane redistributes excitation energy between PSI and PSII via state 1–state 2 transitions, which involve the phosphorylation-dependent migration of LHCII between photosystems and constitute a quick acclimation process [49]. Reduced *α* and increased *β* and *β*/*α* − 1 values throughout our experiment showed that the NaCl treatment upset the equilibrium of excitation energy distribution between the two photosystems in tomato leaves (Figure 5). Higher excitation pressure on PSII (1 − *qP*) as a result of this imbalance may have resulted in reversible deactivation of reaction centers and structural damage to PSII and thylakoid membranes, which would have hindered electron transport and limited photosynthesis’ overall efficiency. Meanwhile, the energy devoted to non-photochemical dissipation (*Ex*), thermal dissipation (*D*), and thermal dissipation rate (*D*_rate_) increased significantly, while the absorbed light energy allocated to the photochemical reaction of PS II (*P*) (*P*), photochemical rate (*P*_rate_), and electron transport rate (*J*_F_) decreased significantly (Figure 6 and Figure 7). The findings imply that NPQ-dependent thermal dissipation mechanisms were augmented to mitigate PSII photodamage, while this alteration also signifies a diminished ability of PSII reaction centers to harness absorbed light energy. Consequently, the salt stress caused a considerable rise in the leaf photosynthetic functional limitation value (*L*_PFD_) (Figure 6). It is also suggested that MT shortage decreases the regulating ability of energy redistribution between the two photosystems since the application of p-CPA, an inhibitor of MT production, worsened the imbalance of excitation energy distribution between PSI and PSII (Figure 5). As demonstrated by higher Fv/Fm values, exogenous MT, on the other hand, enhanced the openness of PSII reaction centers (*qP*) under both NaCl and NL treatments, assisted in balancing the excitation energy allocation between PSI and PSII, and encouraged energy flow toward photochemical reactions (*P*). These actions reduced excess energy (*Ex*) and mitigated PSII damage. Thus, under salt stress, MT helps to maintain appropriate excitation energy partitioning, which eventually improves tomato seedlings’ light energy usage efficiency.

The three main stages of the Calvin cycle—carbon fixation (carboxylation), carbon reduction, and regeneration of ribulose-1,5-bisphosphate (RuBP)—are responsible for fixing carbon dioxide (CO₂) in C₃ plants [21]. Maintaining the activity of important Calvin cycle enzymes and Rubisco carboxylation efficiency is one of the most important ways to improve photosynthesis in the face of abiotic stress, as several studies have shown [50]. In order to improve salt tolerance, Li et al. [51] found that exogenous sodium nitroprusside increased the activity and transcript levels of important Calvin cycle enzymes, which in turn improved the photosynthetic carbon absorption capability of tomato seedlings under salt stress. MT may control the Calvin cycle, according to our earlier work’s hypothesis, although the underlying processes are yet unknown [19]. To elucidate the regulatory role of MT on Calvin cycle function, we evaluated the activities of several important Calvin cycle enzymes in the current study, including the initial and total activity of Rubisco, phosphoglycerate kinase (PGK), glyceraldehyde-3-phosphate dehydrogenase (GAPDH), fructose-1,6-bisphosphatase (FBPase), fructose-bisphosphate aldolase (FBA), transketolase (TK), sedoheptulose-1,7-bisphosphatase (SBPase), and Rubisco activase (RCA). Additionally, we evaluated the expression of nine related genes. Rubisco is the enzyme that limits the rate of carbon uptake during photosynthesis. Its activity directly affects the effectiveness of CO₂ assimilation and catalyzes the first phase of CO₂ fixation by mixing RuBP with CO₂ to generate two molecules of 3-phosphoglycerate (PGA) [52]. Coordinated expression of the genes RbcS and RbcL, which encode the small and large subunits of Rubisco, respectively, controls the structure and function of the Rubisco holoenzyme. The physiological activation of Rubisco is facilitated by RCA, and the carboxylation efficiency of Rubisco is greatly influenced by its activity. In this investigation, salt stress considerably decreased the expression levels of RbcL, RbcS, and RCA genes and significantly decreased both initial and total Rubisco activity as well as RCA activity (Figure 8 and Figure 9). However, exogenous MT application restored these gene expression levels and enzymatic activities under salt stress, indicating that MT increases the formation of assimilatory power (NADPH and ATP) during the light reactions by upregulating RCA activity and activating Rubisco, which in turn improves CO₂ carboxylation efficiency and photosynthetic electron transport. Two important enzymes in the Calvin cycle’s carbon reduction phase are PGK and GAPDH. GAP serves as both a product of chloroplast photosynthesis and a precursor for the synthesis of ribulose 5-phosphate. The expression of its genes directly influences the efficiency of carbon uptake during photosynthesis. Consequently, GAPDH expression is pivotal in modulating the efficacy of carbon assimilation product transport [53]. According to our research, MT dramatically increased the enzymatic activities and expression levels of PGK and GAPDH under salt stress (Figure 8 and Figure 9). This helped the Calvin cycle function and reduced the feedback inhibition brought on by photosynthate buildup. Regeneration of RuBP is dependent on assimilatory power (ATP and NADPH) generated during the light reactions and involves downstream enzymes of the Calvin cycle. In the RuBP regeneration phase, the enzymes SBPase [54], FBPase [55], TK [56], and FBA [57] are crucial. Photosynthetic efficiency and carbohydrate buildup are influenced by FBPase activity. SBPase controls the flow of carbon into the Calvin cycle at the branching point between the assimilation and regeneration stages. Glyceraldehyde-3-phosphate (G3P) and dihydroxyacetone phosphate (DHAP) are produced when fructose-1,6-bisphosphate (FBP) is broken down by FBA. Plants need these responses in order to respond to abiotic stress [58]. Our findings demonstrated that exogenous MT considerably reduced the inhibitory effects of salt stress on SBPase, FBPase, FBA, and TK activities and transcript levels (Figure 8 and Figure 9). The protective effects of MT were, however, diminished by the administration of p-CPA, an inhibitor of MT production. This suggests that MT promotes RuBP regeneration under salt stress, which in turn boosts the carboxylation efficiency of Rubisco in tomato leaves. In conclusion, MT mitigates the reduction in carbon assimilation capacity brought on by salt stress by increasing the activities and gene expression of Calvin cycle enzymes, reducing feedback inhibition of photosynthetic products, and facilitating Calvin cycle operation under salt stress.

## 5. Conclusions

This work demonstrates that exogenous melatonin (MT) enhances photosynthetic performance and salt stress tolerance in tomato seedlings through multiple interconnected mechanisms. MT specifically offers critical support for effective light energy absorption by preserving chlorophyll levels and adjusting the carotenoid-to-chlorophyll ratio. Concurrently, MT markedly enhances the photochemical efficiency of photosystem II (PSII), facilitates the distribution of excitation energy between PSII and photosystem I (PSI), and diminishes energy dissipation through non-photochemical pathways, thus optimizing the efficiency and distribution of light energy utilization. Moreover, MT augments the CO₂ assimilation capacity by upregulating the activities and gene expressions of pivotal enzymes in the Calvin cycle, thereby facilitating carbon fixation and RuBP regeneration, and further mitigating the detrimental effects of salt stress through the photosynthetic mechanism. MT therapy significantly enhanced the relative growth rate and biomass accumulation of tomato seedlings subjected to salt stress conditions. It is notable that the inhibition of MT synthesis by p-CPA markedly reduced the salt tolerance of plants, further substantiating the crucial regulatory function of MT in the plant response to salt stress. This work offers significant insights into the adaptation of MT to plant stress and its potential use in improving the photosynthetic efficiency and production of crops in saline–alkali conditions.

## Data Availability

The original contributions presented in the study are included in the article; further inquiries can be directed to the corresponding author.

## Supplementary Materials

The following supporting information can be downloaded at: https://www.mdpi.com/article/10.3390/plants14121785/s1, Table S1. Primer sequences used for qRT-PCR analysis.
