# Supplementary material for: Melatonin Improves Salt Tolerance in Tomato Seedlings by Enhancing Photosystem II Functionality and Calvin Cycle Activity
Source: Plants (Basel). 2025 Jun 11;14(12):1785. doi: 10.3390/plants14121785 (PMC12197095; doi:10.3390/plants14121785)
Supplement: Supplementary file 1 [file plants-14-01785-s001.zip › plants-3634725-supplementary.pdf]

Table S1. Primer sequences used for qRT-PCR analysis.

Primer Name	Sequence (5'→3')	Product Length (bp)	Gene name
Actin-F<br>Actin-R	TGGTCGGAATGGGAAAG<br>CTCAGTCAGGAGAACAGGGT	191	*Actin* (reference gene)
RbcL-F<br>RbcL-R	CTGTATGGACCGATGGACTTAC<br>AAGGTCTAAAGGGTAAGCTACATAAG	119	*Rubisco large subunit*
RbcS-F<br>RbcS-R	TGAGACTGAGCACGGATTTG<br>TTTAGCCTCTTGAACCTCAGC	142	*Rubisco small subunit*
PGK-F<br>PGK-R	ACTCTTGTTAGCCATTTCAGTTTGT<br>ACCCTAAGAAGAATTCCAGAACA	93	*3-Phosphoglycerate kinase*
GADPH-F<br>GADPH-R	ACTCTGGTATATGTGTTACTC<br>AGGGAAGCAAGATTACTAAA	83	*Glyceraldehyde-3-phosphate dehydrogenase*
FBPase-F<br>FBPase-R	AATTTCCATCTCTTCCCCACC<br>TCGGTTTCTTGATCTGTGCTG	139	*Fructose-1,6-bisphosphatase*
FBA-F<br>FBA-R	ACATTCCGGCTCTTTTCAAAC<br>AGACCAACCCATTACAAGATCC	125	*Fructose 1,6-bisphosphate aldolase*
TK-F<br>TK-R	TTGGAGAAGATGGACCTA<br>GTGTCTTATTCTTGAGGATTG	141	*Transketolase*
SBPase-F<br>SBPase-R	AGAAATACACCTTGAGATACACCG<br>TCAAGAATCCTAACGGTGCC	150	*Sedoheptulose-1,7-bisphosphatase*
RCA-F<br>RCA-R	TTGGACGGATTCTACATCGC<br>CTCCCCAAACACCCAAAATAAG	204	*Rubisco activase*
